# Modulation of Akt and ERK1/2 Pathways by Resveratrol in Chronic Myelogenous Leukemia (CML) Cells Results in the Downregulation of Hsp70

**DOI:** 10.1371/journal.pone.0008719

**Published:** 2010-01-14

**Authors:** Soumyajit Banerjee Mustafi, Prabir K. Chakraborty, Sanghamitra Raha

**Affiliations:** Crystallography and Molecular Biology Division, Saha Institute of Nuclear Physics, Kolkata, India; University of Washington, United States of America

## Abstract

**Background:**

Resveratrol is known to downregulate the high endogenous level of Heat shock protein 70 (Hsp70) in Chronic Myelogenous Leukemia (CML) K562 cells and induce apoptosis. Since Heat Shock Factor 1 (HSF1) controls transcription of Hsp70, we wanted to probe the signaling pathways responsible for transcriptional activation of HSF1.

**Methodology/Principal Findings:**

Cells exposed to 40µM Resveratrol rapidly abolished serine473 phosphorylation of Akt and significantly reduced its kinase activity. Inactivation of Akt pathway by Resveratrol subsequently blocked serine9 phosphorylation of Gsk3β. Active non-phosphorylated Gsk3β rendered HSF1 transcriptionally inactive and reduced Hsp70 production. Blocking PI3K/Akt activity also demonstrated similar effects on Hsp70 comparable to Resveratrol. Inactivation of Gsk3β activity by inhibitors SB261763 or LiCl upregulated Hsp70. Resveratrol significantly modulated ERK1/2 activity as evident from hyper phosphorylation at T302/Y304 residues and simultaneous upregulation in kinase activity. Blocking ERK1/2 activation resulted in induction of Hsp70. Therefore, increase in ERK1/2 activity by Resveratrol provided another negative influence on Hsp70 levels through negative regulation of HSF1 activity. 17-allylamino-17-demethoxygeldanamycin (17AAG), a drug that inhibits Hsp90 chaperone and degrades its client protein Akt concomitantly elevated Hsp70 levels by promoting nuclear translocation of HSF1 from the cytosol. This effect is predominantly due to inhibition of both Akt and ERK1/2 activation by 17AAG. Simultaneously treating K562 with Resveratrol and 17AAG maintained phosho-ERK1/2 levels close to untreated controls demonstrating their opposite effects on ERK1/2 pathway. Resveratrol was found not to interfere with Bcr-Abl activation in K562 cells.

**Conclusion/Significance:**

Thus our study comprehensively illustrates that Resveratrol acts downstream of Bcr-Abl and inhibits Akt activity but stimulates ERK1/2 activity. This brings down the transcriptional activity of HSF1 and Hsp70 production in K562 cells. Additionally, Resveratrol can be used in combination with chemotherapeutic agents such as 17AAG, an Hsp90 inhibitor reported to induce Hsp70 and hence compromise its chemotherapeutic potential.

## Introduction

Cells are armed with various mechanisms which counteract stress to maintain cellular homoeostasis when challenged with subtle to acute changes in the physical, cellular or intracellular environment. Such a stress response helps the cell to evade apoptotic cell death and survive. Heat Shock Proteins (Hsps) are a family of stress proteins, both constitutive and inducible, primarily with chaperoning properties that help the cell to maintain cellular protein homeostasis and also to escape apoptosis under diverse forms of stress, from heat to alkalosis. In normal cells Hsp gene transcription is under the strict regulation of transcription factors belonging to the heat shock factor (HSF) family that ensure prompt switching on of transcriptional activity during stress and equally important post-stress switch off during recovery [1]. In cases where this co-ordination of HSF1 activation, stress response and post recovery deactivation mechanism is not well co-ordinated, the Hsps become highly overexpressed. This may render the cells anti-apoptotic and may eventually lead to malignancy. In fact a large number of malignancies have been linked to the overexpression of Hsps, in particular Hsp27, Hsp70 and Hsp90 [2].

With the growing evidence of an important role for Hsps in cancer, researches on chemotherapeutic strategies targeting Heat shock proteins 90 and 70 have gained momentum over the past few years [3]. Although the bigger goal remained the same, research strategies varied ranging from designing targeted small molecule drugs like 17AAG, against Hsp90 to introduction of peptide–Hsp70 complexes as anti-cancer vaccines in immunotherapy approach [4]–[6].

Another new approach in treating cancer is the awareness of the important pharmacological action of many natural products such as lycopene, curcumin, capsaicin, EGCG, Resveratrol etc. [7]. Current research has established important roles for the natural products in receptor binding, modulation of pro-survival signaling pathways and targeting various oncoproteins without affecting normal cells [8]. Epidemiological studies have demonstrated that the consumption of fruits, soybean and vegetables is related to the reduction in risk of several types of cancers [9]. Current findings on Resveratrol suggest that it has role in neuro-protection, cardio-protection and also has chemopreventive and chemotherapeutic properties in solid and haematologic malignancies [10]–[12]. The uniqueness of Resveratrol lies in its ability to bind multiple targets, many of which are closely related to the patho-physiological causes of disease. Resveratrol activates important transcription factors such as NFκB, STAT3, HIF-1α, β-catenin and antiapoptotic gene products which include Bcl-2, Bcl-X(L), and inhibitors of apotosis (IAP such as survivin) [13]. Resveratrol down-regulated Akt, mitogen-activated protein kinases, and Wnt signaling pathways [14]. In our previous study we have firmly established that Resveratrol can target Hsp70 to induce apoptosis in Chronic Myelogenous Leukemia (CML) [11]. CML cells showed high level of endogenous Hsp70 both constitutive and inducible which made the cells resistant to apotosis by the chemotherapeutic drug Imatinib Mesylate and Nilotinib [15].

Chronic myelogenous leukemia (CML) is associated with a characteristic chromosomal translocation between chromosomes 9 and 12, called the Philadelphia chromosome. This gene directs the expression of a mutant, constitutively active tyrosine kinase called Bcr-Abl that activates a large number of proteins controlling cell cycle and inhibiting DNA repair, resulting in speeding up of cell division and incorporation of further genetic abnormalities [16]. Hence the standard care for treating CML is to target Bcr Abl tyrosine kinase by the pharmacological inhibitors Imatinib or more potent Dasatinib and Nilotinib [17]. However resistance or intolerance to these tyrosine kinase inhibitors is often encountered and therefore warrants the search for alternate targets [18].

Overexpression of the heat-shock protein 70 is associated with Imatinib resistance in chronic myeloid leukemia [15].We have previously reported that Resveratrol alters sub cellular localization, transcriptional activity, and promoter binding of the HSF1 that in turn downregulates gene expression of hsp70 [11]. However how Resveratrol modulates HSF1 activity remains unexplored. HSF1 transcriptional activity largely depends on the phosphorylation at specific residues. Phosphorylation on residues Ser303 and 307 by GSK3β and p42/44MAPK(ERK) respectively has been reported to have a negative regulatory effect on HSF1 activation [19], [20]. Recent evidence also claims that negative regulation of HSF1 can be accomplished by its acetylation at K80 [21]. Therefore, HSF1 seems to be at the crossroads of different signaling pathways and the heat shock response. Extracellular signal-regulated kinase (ERK) 1/2 and Akt kinase are reported to be constitutively active in the chronic phase of CML, blast crisis of CML, and the CML-derived K562 cell line [22]. MEK1/2 inhibitors sensitize K562 and LAMA cells to the dual Abl/Src inhibitor BMS-354 825 (Dasatinib) [23]. In our present study we have tried to decipher the signaling pathways that contribute to the constitutive activation of HSF1 in K562 cells.

17AAG, an Hsp90 inhibitor is reported to induce Hsp70 [24] and hence compromise its chemotherapeutic potential. We have previously shown that Resveratrol can potentiate the antiapoptotic efficacy of 17AAG in K562 when used in combination [11]. Akt is a substrate of the chaperone Hsp90. 17AAG downregulated Akt by virtue of its inhibitory role on Hsp90. Resveratrol is also known to downregulate Akt activity [25]–[27]. Even though both Resveratrol and 17-AAG downregulate Akt activity their effects on Hsp70 expression are totally dissimilar. Our present study addresses this contradiction by elucidating the signaling pathways involving Akt, Gsk3beta and p42/44MAPK. We hereby decipher a comprehensive signaling pathway to firmly establish the role of Resveratrol in abrogating the Hsp70 levels in K562 cells.

## Materials and Methods

### 2.1 Materials

Resveratrol was purchased from Calbiochem, UK. Antibodies against Phospho Akt (ser473, CST) Akt (CST), Hsp72 (Abcam), P-Gsk3β (Abcam), Erk1/2 (Sigma), P-ERK1/2 (T-202, Y204, CST) were purchased from established vendors. Monoclonal mouse anti-actin and mouse anti-Hsp70 antibodies were purchased from BD Pharmingen. Rabbit anti-HSF1 was obtained from Abcam and secondary antibodies were obtained from Calbiochem. MBP and synthetic peptide Gsk3β were obtained from Cayman and Calbiochem respectively.

### 2.2 Cell Culture and Drug Treatment Conditions

Human chronic myeloid leukemia cells K562 obtained from the Cell Repository of National Centre for Cell Science, Pune, India were maintained in exponential growth in RPMI-1640 medium supplemented with 10% heat-inactivated fetal bovine serum, 100 units/mL penicillin and 100 µg/mL streptomycin in a humidified atmosphere of 5% CO_2_ at 37°C. Resveratrol, Wortmanin, 17AAG, SB216763, U0126 or LiCl were dissolved in dimethylsulfoxide or water (only for LiCl) at a concentration of 40mM, 1mM, 2.5mM, 5mM, 10mM and 1M respectively and added to cells at the indicated concentrations and time.

### 2.3 Western Blot

Protein-extracts, 20–50 µg, were first separated by 10% sodium dodecyl sulfate-polyacrylamide gel electrophoresis (SDS-PAGE) and were then transferred onto polyvinylidene fluoride membrane. After blocking with 5% bovine serum albumin (BSA), membranes were incubated overnight at 4°C with respective primary antibodies. The membranes were incubated with 1∶5,000 dilutions of the appropriate peroxidase-conjugated secondary antibodies and or alkaline phosphatase-conjugated antibodies and developed for detection by chemiluminescence or colorimetry. Western blots were scanned and the bands were quantified by using NIH ImageJ software.

### 2.4 Immunoprecipitation and Radioactive Kinase Assay

Equilibration of bead with IP buffer: 40µl–60µl of 1∶1 protein agarose A was taken and centrifuged at 10000g for 1 min and the supernatant was aspirated. The beads were suspended in IP buffer and centrifuged at 1000g for 1 min. After equilibration with IP buffer beads were washed and suspended in 50µL IP buffer. 1µg of required antibody was added (ERK1/2 or Akt) to 1mg of treated and untreated lysate and incubated overnight at 4°C in spin wheel. The equilibrated beads were added to the protein/antibody homogenate on the next day and incubated at 4°C for 2 ½ h in a spin wheel. The beads were collected by centrifugation, washed and suspended in 15 µL IP buffer to be used for radioactive or nonradioactive kinase assay. Radioactive assay for Erk1/2 was performed using 10 µg of MBP with 9 µl of final immunoprecipitate bead in 15 µl reaction mix respectively containing 50 mM Tris–Cl pH 8.0, 10 mM MgCl_2_, 50 µM ATP and 5 µCi of [γ-^32^P] ATP (3000 Ci/mmol). The reactions were carried out for 15 min at 25°C after which they were stopped by adding 4× sample loading buffer. The samples were subjected to SDS-PAGE, the gels were dried and exposed at −80°C as required on an X-ray film and developed. Non radioactive assay for Akt used similar protocol with only the radioactive ATP being replaced by 200mM of cold ATP and Gsk3β being used as substrate. After the kinase reaction, normal Western blot analysis was performed with P- Gsk3β (serine9) primary antibody. The films were scanned and quantified using Image J software.

### 2.5 Fluoroscence Microscopy

K562 cells were fixed in 4% p-formaldehyde in phosphate buffered saline(PBS) and incubated at room temperature for 30 min. The cells were washed twice with chilled PBS (washed at 6000g for 5 min. at 4°C). The cells were permeabilized with 0.2% Triton×100 for 10 min at 37°C, followed by blocking with 3% BSA in PBS for 2h at room temperature. The cells were incubated with desired antibody solution (Phospho Erk1/2 or Phospho Akt) in PBS with 1% BSA overnight and then incubation with secondary antibody conjugated with FITC was done for 1h at room temperature. The cells were washed in PBS several times and then loaded on slide with 10uL of DAPI (Vecta Shield procured from Vector Laboratory Inc USA). Coverslips were sealed of with nail-polish and viewed at 40× magnification using 535nm emission wavelength for FITC and 445nm for DAPI in Zeiss fluorescence microscope.

### 2.6 Statistical Analysis

Student's t-test was used to calculate statistical significance of the data.

## Results

### 3.1 Resveratrol Downregulates the Akt/PKB Phosphorylation and Activity

**Figure 1:**
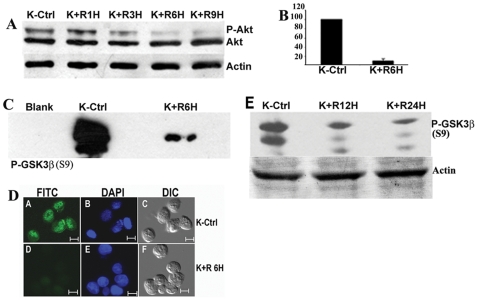
Alteration in Akt activation in response to Resveratrol: (A) Akt phosphorylation shown in immunoblot. In the upper panel phospho serine^473^ Akt immunoblot is displayed. In the middle panel Akt and in the lower panel Actin immunoblots are displayed to indicate loading controls. K-Ctrl – untreated K562 cells; K+ R1H- K562 cells treated with Resveratrol for 1h; K+ R3H- K562 cells treated with Resveratrol for 3h; K+ R6H- K562 cells treated with Resveratrol for 6h; K+ R9H- K562 cells treated with Resveratrol for 9h. **(B) The panel displays the ratio (mean±SD) of the P-Akt protein/Akt protein band densities of K562 control cells (K-Ctrl) and 6h Resveratrol treated cells (K+ R6H) from three experiments.** **(C) Results of Akt Kinase assay are displayed as immunoblot.** Total Akt was immunoprecipitated from Resveratrol treated or untreated cell lysates (containing equal amount of total protein) and kinase assay was performed with equal amount of the precipitated beads and GSK3β synthetic peptide and ATP as substrates and P- GSK3β was detected by Western Blot using anti serine^9^ GSK3β antibody. K-Ctrl – untreated K562 cells; K+ Res (6H) - K562 cells treated with Resveratrol for 6 h. **(D) Cellular localization of phospho serine^473^ Akt by fluorescence microscopy.** A: Untreated K562 cells (K-Ctrl) processed for visualization of P-Akt with secondary antibody FITC conjugate.; B: Untreated K562 cells processed for visualization of DNA using specific stain DAPI; C-: DIC images of the cells; D: K562 cells treated with Resveratrol for 6 h and processed for visualization of P-Akt with secondary antibody FITC conjugate.; .E: K562 cells treated with Resveratrol for 6 h and processed for visualization of DNA using specific stain DAPI; F: DIC images of the cells. **(E) Phospho-GSK3 immunoblot.** Upper panel shows phospho-GSK3β and lower panel shows actin as loading control. K-Ctrl – untreated K562 cells; K+ R12H - K562 cells treated with Resveratrol for 12h; K+ R24H - K562 cells treated with Resveratrol for 24h. The bar represents 10µm. All the blots and micrographs are representative of two to three sets of separate experiments. Concentration of Resveratrol is 40µM. Details of the experiments are described in section-2.

We first confirmed if Resveratrol has any significant role in altering the Akt activity. 40µM Resveratrol significantly curtailed the phosphorylation (Ser 473) of Akt by >25% within 1h of treatment of K562 cells (Fig. 1A). This effect was further bolstered at later time points up to 9h with a significant drop of around 10 fold (P<0.05) registered at 6h (Fig 1B). Phosphorylation of Akt at 473 serine residue is directly linked to the kinase activity of the protein. Therefore a kinase assay was performed to determine if Resveratrol could also efficiently alter the catalytic activity of Akt. Exposure to 40µM Resveratrol for 6 h resulted in a significant drop (84%±7%) in the kinase activity of Akt (Fig. 1C). Resveratrol thus effectively compromised the phosphorylation and activity of one of the major oncoprotein, Akt, highly active in chronic myelogenous leukemia. Fig. 1D demonstrates that phosphorylated Akt is predominantly located in the nucleus of K562 cells but in K562 cells treated with Resveratrol for 6h phosphorylated Akt could be visualized neither in the nucleus nor in other parts of the cell. Akt in both cases (untreated control and Resveratrol treated) is almost equally distributed in the cytoplasm and nucleus (Fig. S1). Fig. 1E shows the phosphorylation status of the Akt substrate GSK-3β (serine9) which is markedly phosphorylated in K562 cells but the phosphorylation is diminished drastically upon Resveratrol exposure.

### 3.2 Effects of Blocking Akt Activation Pathway by PI3-Kinase Inhibitor Wortmanin

**Figure 2:**
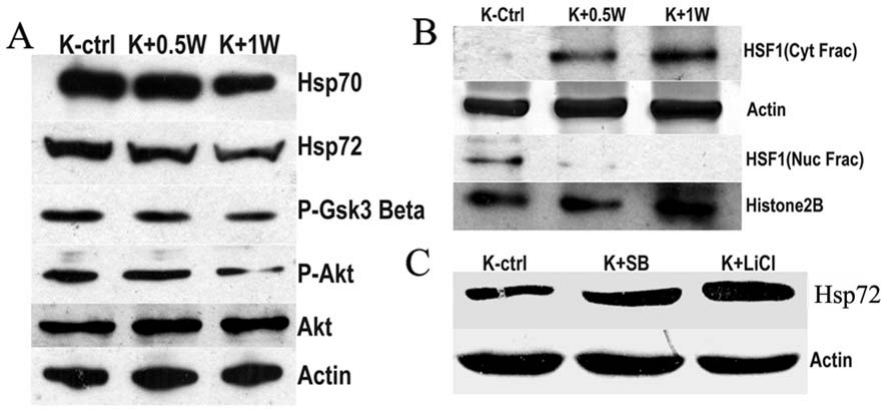
Effects of Wortmanin, SB216763 and LiCl treatment on K562 cells: (A) Uppermost panel depicts the protein level of Hsp70 by Western blot. Second panel from top depicts Hsp72 by Western blot. The third panel from top shows phosho serine^9^ GSK3β by Western blot. The fourth panel from top shows phospho serine^473^ Akt levels by Western blot. The fifth and the sixth panels from top displays Akt and actin protein levels by Western blot to denote loading controls. K-Ctrl - control K562 cells; K+0.5W- K562 cells treated with 0.5µM Wortmanin for 24h; K+1W- K562 cells treated with 1µM Wortmanin for 24h. **(B) Localization of HSF1 after Wortmanin exposure.** Uppermost panel represents cytosolic localization of HSF1 after 24h treatment with different concentrations of Wortmanin. The second panel from top represents actin immunoblots to indicate loading control for cytosolic fraction. The third panel from top represents nuclear localization of HSF1 after 24h treatment with different concentration of Wortmanin. The fourth panel from top represents Histone2B immunoblots to indicate loading control for nuclear fraction. K-Ctrl - control K562 cells; K+0.5W- K562 cells treated with 0.5µM Wortmanin; K+1W- K562 cells treated with 1µM Wortmanin. **(C) Effect of GSK3β inhibitors on Hsp72 levels.** Upper panel represents immunoblot of Hsp72 and lower panel represents actin immunoblot to indicate loading control. K-ctrl - K562 cells left untreated; K+ SB – K562 cells treated with GSK pharmacological inhibitor 5 µM SB216763 for 24h; K+ LiCl - K562 cells treated with 10mM LiCl for 24 h. All blots are representative of two separate experimental sets.

In our previous study we have conclusively established that Resveratrol downregulated Hsp70 levels and induced apoptosis in K562 cells [11]. However it was not exactly known how Resveratrol accomplished this effect on Hsp70. Exposure to Wortmanin (0.5 µM and 1µM), a potent pharmacological inhibitor for PI3K, heavily curtailed the phosphorylation of (serine 473)Akt and (serine9)Gsk3β(Fig. 2A) in K562 cells. Hsp70 levels and stress inducible form Hsp72 showed subsequent drop in protein levels on exposure to Wortmanin (Fig 2A). Since Resveratrol blocked Akt activation it was essential to demonstrate if Akt activation pathway was involved in modulating the subcellular distribution and activation of HSF1, the transcription factor responsible for expression of hsp70 gene. Measuring the levels of cytosolic and nuclear fraction of HSF1 by Western blot analysis, it was found that exposure to 0.5µM Wortmanin for 24h appreciably augmented the cytosolic localization of HSF1 which showed a further increase at 1µM concentration. In contrast Wortmanin at both the above concentrations clearly decreased the levels of nuclear HSF1 (Fig 2B). Wortmanin treatment also lowered the phosphorylated form of Gsk3β levels, which is the catalytically inactive form. This is quite understandable as Gsk3β is a substrate for phosphorylation by Akt. Therefore it was interesting to see if Gsk3β inactivation could result in upregulation of Hsp70 in K562 cells. Blocking Gsk3β by two different inhibitors LiCl (10mM) and SB216763 (5 µM) resulted in considerable increase in Hsp72 levels over untreated control after 24h of treatment (Fig 2C). LiCl exerted a stronger increase of 2.5 fold whereas SB216763 produced a moderate increase of 1.7 fold.

### 3.3 Upregulation of ERK1/2 Activity by Resveratrol

**Figure 3:**
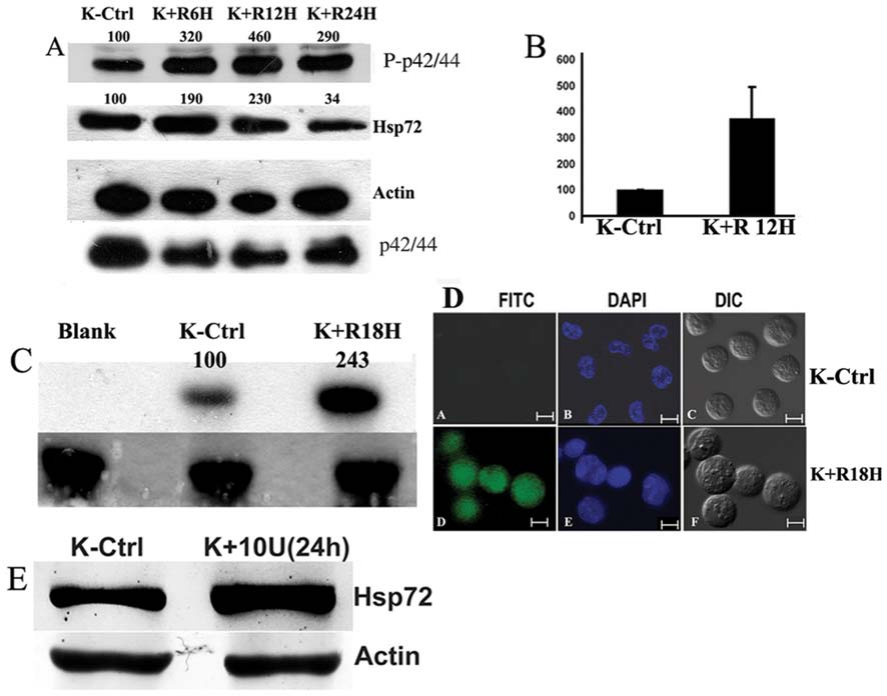
Alteration in p42/44 ERK activation in response to Resveratrol: (A) Effect of Resveratrol on ERK phosphorylation. In the upper panel phospho threonine^202^/phospho tyrosine^204^ p42/44 ERK immunoblot is displayed. In the middle panel Hsp72 immunoblot is displayed and in the lower panels Actin immunoblot and ERK1/2 immunoblots are displayed to indicate loading controls. At the top of the upper and middle panels band densities of P-ERK and Hsp70 bands are given as percentage of control values taken as 100. K-Ctrl – untreated K562 cells; K+ R6H- K562 cells treated with Resveratrol for 6 h; K+ R12H- K562 cells treated with Resveratrol for 12 h; K+ R24H- K562 cells treated with Resveratrol for 24 h; **(B) The figure displays the ratio (mean±SD) of the P-ERK1/2 protein/actin protein band densities of K562 control cells (K-Ctrl) and 12h Resveratrol treated cells (K+ R12H) from three experiments.** **(C) ERK1/2 Kinase Assay.** Results of ERK1/2 Kinase assay is displayed as autoradiograph. Total p42/44 ERK was immunoprecipitated (IP) from Resveratrol treated or untreated cell lysates (containing equal amount of total protein) and kinase assay was performed with equal amount of the precipitated beads and purified MBP and radioactive ATP as substrates. The figures above the panel represents band intensity as percentage of control values taken as 100. Blank: Kinase assay buffer without the IP beads; K-Ctrl – untreated K562 cells; K+ Res (18H) - K562 cells treated with Resveratrol for 18h. The lower panel depicts coomassie stained bands to denote equal amount of MBP used for the kinase reaction. **(D) Cellular localization of phospho threonine^202^/phospho tyrosine^204^ p42/44 ERK by fluorescence microscopy.** A: Untreated K562 cells (K-Ctrl) processed for visualization of P-ERK with secondary antibody FITC conjugate.; B: Untreated K562 cells processed for visualization of DNA using specific stain DAPI; C-: DIC images of the cells; D: K562 cells treated with Resveratrol for 18 h and processed for visualization of P-ERK with secondary antibody FITC conjugate.; E : K562 cells treated with Resveratrol for 18h and processed for visualization of DNA using specific stain DAPI; F: DIC images of the cells. The bar represents 10µm. **(E) Effect of ERK inhibitor on Hsp72 levels.** Upper panel depicts Hsp72 and the lower panel shows actin immunoblots to represent protein loading. K-ctrl - K562 control cells; K+ 10U (24H) - K562 cells treated with pharmacological inhibitor against MEK, U0126 (10 µM) for 24h. All the blots and micrographs are representative of two to three sets of separate experiments. Concentration of Resveratrol is 40µM. Details of the experiments are described in section-2.

Resveratrol at 40 µM concentration was found to induce hyperphosphorylation of ERK1/2 in a time dependent manner (Fig 3A). The induction of Phosphorylation of ERK1/2 was most prominent at 12h which recorded almost 3.2 fold increase over control when normalized to internal loading control actin (Fig. 3B). Hsp72 (stress induced Hsp70) also showed a simultaneous decline on Resveratrol exposure up to 24h (Fig 3A). However the decline in Hsp72 status was most prominent at 12 and 24h (drop of ∼40%) with almost no appreciable change taking place up to 6h. Resveratrol was shown to induce ERK1/2 phosphorylation in a time dependent manner. Whether this hyper-phosphorylated status of ERK1/2 also reflected in the kinase activity of the protein was important to understand. In vitro radioactive kinase assay was performed using radiolabelled ATP and MBP as substrates of the ERK1/2 protein immunoprecipitated from the whole cell extracts either exposed to 40µM resveratrol for 18h or left untreated. High level of radiolabelled MBP (P-MBP) was detected by autoradiography for cells treated with Resveratrol (Fig 3C) compared to that of untreated control. Resveratrol brought about almost a 2.4 fold increment in kinase activity. When cells were treated with P-ERK1/2 antibody and visualized by FITC conjugated secondary antibody, Resveratrol was found to induce ERK1/2 phosphorylation as K562 cells treated with Resveratrol showed distinct green florescence compared to untreated control (Fig. 3D). P-ERK1/2 was found to be distributed more abundantly in the nucleus. Resveratrol was found to simultaneously induce ERK1/2 phosphorylation and downregulate Hsp72 levels in K562 cells. However it was important to understand if there was any correlation between these two events. Therefore ERK1/2 activity was blocked using the pharmacological inhibitor against MEK, U0126. Blocking activation of ERK1/2 by growing cells in presence of inhibitor for 24h resulted in simultaneous up-regulation in the Hsp72 levels by almost 1.8 fold (Fig 3E).

### 3.4 17AAG Treatments Blocks Akt but Boosts the Hsp 70 Levels

**Figure 4:**
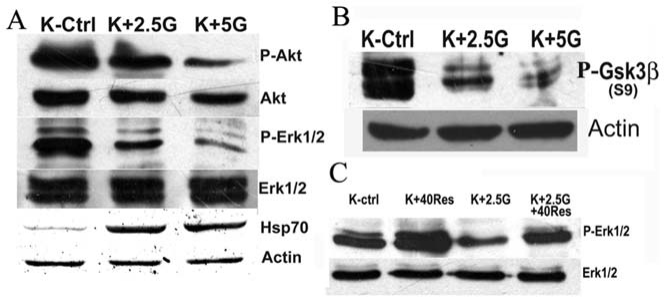
Effects of 17AAG and Resveratrol treatment on K562 cells: (A) Effects of 17-AAG on phosphorylations of Akt and ERK 1/2, and protein levels of Hsp70. Uppermost panel depicts phospho serine^473^ Akt levels by Western blot. Second panel from top depicts Akt by Western blot to represent protein loading. The third panel from top indicates phospho threonine^202^/phospho tyrosine^204^ p42/44 MAPK immunoblot. The fourth panel from top shows p42/44 MAPK by Western blot to represent corresponding loading control. The fifth panel from top displays Hsp70 protein levels and sixth panel depicts actin protein levels by Western blot. K-Ctrl - control K562 cells; K+2.5G- K562 cells treated with 2.5µM 17AAG for 24h; K+5G- K562 cells treated with 5µM 17AAG for 24h. **(B) Effect of 17-AAG on GSK3β.** The upper panel shows immunoblots of phospho serine^9^ GSK3β in response to treatment with different dose concentration of 17AAG for 24h. The lower panel represents actin immunoblots. K-Ctrl - control K562 cells; K+2.5G- K562 cells treated with 2.5µM 17AAG for 24h; K+5G- K562 cells treated with 5µM 17AAG for 24h. **(C) Effects of a combination of 17-AAG and Resveratrol on phosphorylation status of ERK1/2.** The top panel indicates phospho threonine^202^/phospho tyrosine^204^ p42/44 ERK immunoblot and the bottom panel indicates p42/44 ERK to represent protein loading.. K-Ctrl - untreated K562 cells; K+ 40Res - K562 cells treated with 40µM Resveratrol for 24h; K+2.5G- K562 cells treated with 2.5µM 17AAG for 24h; K+2.5G+40Res - K562 cells treated simultaneously with 2.5µM 17AAG and 40µM Resveratrol for 24h. All blots are representative of two to three independent experimental sets.

The findings above indicated the important roles of Akt and ERK1/2 activation in down regulation of Hsp70. However the question that needs to be addressed is how diminishing Akt activation by17AAG treatment (Fig 4A) could simultaneously result in induction of stress response by sharply augmenting the Hsp70 levels (Fig 4A). 17AAG blocks the chaperoning activity of Hsp90, prompting degradation of its client protein Akt. This is well demonstrated by the dose dependent decline of Akt and phospho serine 473 Akt levels in K562 cells treated with 2.5µM and 5 µM concentration of 17AAG (Fig 4A). Phospho Gsk3β levels also showed a sharp decline with treatment with 2.5 µM 17AAG and this de-phosphorylation of Gsk3β was further augmented at higher concentration of 5 µM 17AAG (Fig 4B). Since down regulation of Akt activity and corresponding Gsk3β activation both are known to be associated with Hsp70 down regulation (Fig 1,2) the effect of 17AAG on ERK1/2 gains importance and holds the key to this apparent puzzle. Exposure to 17AAG induced a distinct de-phosphorylation of ERK1/2 (T-202/Y-204) by several fold (8 fold, P<0.05). When combined with Resveratrol, 17AAG demonstrated an opposite effect on ERK1/2 activation in K562 cells. After simultaneous exposure of K562 to both 40 µM Resveratrol and 2.5 µM 17AAG for 24h the levels of P-ERK1/2 were much higher than that in cells treated with 17-AAG alone and were close to untreated controls (Fig 4C). In comparison, ERK1/2 Phosphorylation status in response to only 40 µM Resveratrol recorded almost 2 fold increase over control (Fig 4C).

### 3.5 Resveratrol Does Not Inhibit Tyrosine Kinase Bcr-Abl in K562

**Figure 5:**
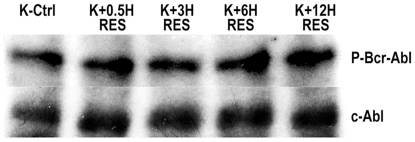
Effects of Resveratrol treatment on phosphorylation of Bcr-Abl. Upper panel depicts phospho tyrosine^177^ Bcr-Abl immunoblots at various time points after 40µM Resveratrol treatment in K562 cells. Lower panel represents c-Abl protein levels by Western blot to indicate protein loading. K-Ctrl - untreated K562 cells; K+0.5H Res – K562 cells treated for 30min with Resveratrol; K+3H Res – K562 cells treated for 3h with Resveratrol; K+6H Res – K562 cells treated for 6h with Resveratrol; K+12H Res – K562 cells treated for 12h with Resveratrol.

Most of the current drugs for Philadelphia chromosome^+^ CML patients undergoing clinical trials are effectively Tyrosine Kinase inhibitors blocking Bcr-Abl activity. Resveratrol acted downstream of Bcr-Abl as evident from Western blot analysis of Phospho Bcr-Abl. K562 cells exposed to 40µM Resveratrol for various time intervals ranging from 30min up to 12h failed to demonstrate any significant alteration of Bcr-Abl phosphorylation status (Fig 5).

## Discussion

Over the last few years Resveratrol have been the subject of extensive research which has elucidated the multifaceted role of this phytochemical from chemotherapeutic/chemopreventive potential to promoter of longevity [28]. The list of molecules targeted by Resveratrol is long and is being continuously updated. But the signaling pathways operative upstream of these final targets are often not well studied. We have previously established the efficacy of Resveratrol as a pro-apoptotic agent in K562 cells, essentially by virtue of its effect on the cellular chaperone Hsp70 [11]. As high endogenous levels of Hsp70 is a pathophysiological feature not only of CML but also of a large number of cancers [29], the downregulation of Hsp70 by Resveratrol in K562 serves as a very effective indicator of the chemotherapeutic potential of Resveratrol. This makes understanding of the signaling pathways modulated by Resveratrol for downregulating Hsp70 more relevant. Resveratrol has been shown to be able to induce apoptosis in Imatinib mesylate-resistant human chronic myelogenous leukemia cell lines [30]. Exaggerated expression of Hsp70 is reported to be the most important protein responsible for Imatinib resistance in CML [15], [30]. There are some reports on the effect of Resveratrol on Akt and MAPK pathways, but the connections between these signaling pathways and Hsp70 have not been thoroughly explored. Resveratrol was found to modulate the function of HSF1 as a transcription factor for Hsp70 in K562 cells [11]. However the role of upstream kinases in controlling HSF1 activity was not properly delved into.

PI3K/Akt pathway has emerged as one of the essential signaling mechanisms in ABL leukemogenesis as its downstream effectors are responsible for propagating the signals to promote myeloid and lymphoid transformation [31]. Resveratrol was able to diminish the levels of the activated form of Akt phosphorylated at ser437 within 3h of incubation and this effect was even more pronounced at 6h. Akt activity was also suppressed as supported by the in vitro Akt substrate phosphorylation experiment (Fig. 1C). The Akt pathway has been the target of many natural compounds like Resveratrol, Curcumin, tea polyphenols etc. for exerting their chemopreventive effects [8]. Phosphorylated Akt is responsible in activating and de-activating phosphorylation of numerous substrates. Blocking of Akt phosphorylation by the PI3K inhibitor Wortmanin indicated that Hsp70 expression is dependent on activated Akt. (Fig, 2A). The phosphorylation based inactivation of GSK3β (ser9) by Akt is also abrogated in presence of Wortmanin (Fig 2A). It is reasonable to assume that GSK3β is more active in the absence of its inhibitory phosphorylation at ser9. Also, the HSF1 translocation to the nucleus was abrogated by Wortmanin at concentration as low as 0.5µM. Thus Akt pathway controls the translocation of HSF1 to the nucleus. The HSF1 activation and translocation to the nucleus is dependent on phosphorylation of its multiple amino acid residues [32]. One of these phosphorylation is contributed by GSK3β at ser 303 that prevents HSF1 to accumulate in the nucleus [33]. Thus the activation of GSK3β in presence of Resveratrol definitely contributes to the blocking of HSF1 translocation to the nucleus and hence Hsp70 expression. We also provided evidence of involvement of GSK3β in the upregulation of Hsp70 by using LiCl, known to be a classical inhibitor of GSK3β and the synthetic specific inhibitor SB216763. In presence of LiCl and SB 216763, K562 cells exhibited higher induction of Hsp70 indicating the process to be governed by GSK3β. Although LiCL is a powerful inhibitor of GSK3β it also inhibits other protein kinases and influences inositol phosphate signaling [34]. Furthermore, LiCl has been shown to stimulate Akt which phosphorylates and inhibits GSK3β [34]. Therefore, LiCl may inhibit GSK3β in different ways and emerge as a less specific but more potent inhibitor of Hsp70 expression possibly through its action on other cellular targets. In contrast, SB216763 is a very specific inhibitor of GSK3β and does not inhibit other protein kinases [34]. As a result, SB216763 may act through a single target and have a more moderate effect on Hsp72 levels.

**Figure 6:**
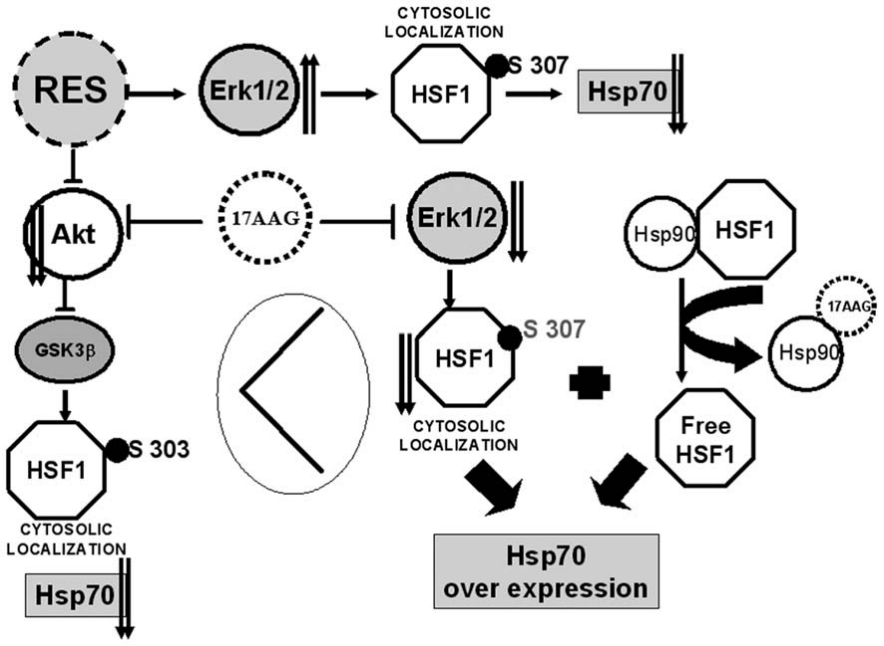
Schematic representation of the signaling pathways operative in Hsp70 regulation in Chronic Myelogenous Leukemia. Akt and ERK1/2 both phosphorylate HSF1 on serines (ERK1/2 on serine 307 and GSK3β on serine 303 to negatively influence HSF1 activity). In fact, the phosphorylation by ERK1/2 at Serine 307 is the priming event and it facilitates the phosphorylation by GSK3β at serine 303 which has vital regulatory influence on HSF1 activity. Resveratrol inhibits Akt but stimulates the activity of ERK1/2. Therefore, Resveratrol is able to influence both the priming and the subsequent inhibitory phosphorylation. 17-AAG inhibits Akt and as a consequence decreases the phosphorylation of GSK3β and makes GSK3β active. However, 17AAG also decreases the activation of ERK1/2. Therefore, the active GSK3β is not able to negatively influence HSF1 activity. 17-AAG binds Hsp90 and releases Hsp90 bound HSF1. Free HSF1 in the absence of negative influence of phosphorylations enters the nucleus and triggers the transcription of Hsp70.

We also checked the effect of Resveratrol on phospho ERK1/2 as this kinase is required for the primary priming of HSF1 at ser 307 so that GSK3β can perform the secondary phosphorylation on HSF1 at ser 303 resulting in a cytoplasmic –inactive form of HSF1 [33]. In presence of Resveratrol there was a considerable increase in the phosphorylated ERK1/2 signal and ERK1/2 activity was also enhanced (Fig 3A–C). The command of phospho ERK1/2 on the suppression of Hsp70 expression in K562 cells was shown by inhibiting ERK1/2 kinase activity with U0126 which resulted in an elevated expression of Hsp72 (Fig. 3E). From these aforesaid results it can be concluded that Resveratrol influences the two kinases namely GSK3β and ERK1/2 (Fig. 6).

These in turn inactivate HSF1 so as to decrease Hsp70 expression in K562 cells in presence of Resveratrol.

The exact mode of action of Resveratrol in connection with inhibition of Akt and stimulation of ERK activity is not apparent at present. However, the literature shows that Resveratrol-induced ERK activation may happen through induction of sirtuin SIRT1 (silent mating type information regulation 2 homolog) by Resveratrol [35]. However, Akt inhibition may take place in a SIRT1 –independent way [35], [36] through disruption of interaction between upstream signaling elements such as PI3 Kinase or Grb2 (growth factor receptor-bound protein 2) with further upstream proteins relaying signals directly from the receptor [36].

While proposing the role of Akt in downregulating Hsp70 by Resveratrol in K562 cells we contradicted the observation of induction of stress response by 17AAG, a drug that is also known to lower Akt levels (Fig. 4A) by virtue of its action on Hsp90. 17AAG was found to reduce ser473 phosphorylation of Akt and ser9 phosphorylation of Gsk3β (Fig. 4A and B) exhibiting virtually similar effects as of Resveratrol on Akt and Gsk3β. But 17 AAG effect on Hsp70 strikingly differed with Resveratrol in the Resveratrol mediated downregulation of Hsp70 as 17 AAG exposure resulted in the upregulation of Hsp70. (Fig 4A). To explain this paradox we looked at the effect of 17AAG on ERK1/2 activation we found that ERK1/2 was markedly dephosphorylated when cells were exposed to 2.5µM and 5µM 17AAG (Fig. 4A). As ERK1/2 plays an extremely important role in negatively influencing HSF1 activity through a priming phosphorylation [33], ERK1/2 inactivation by 17AAG seems to positively influence Hsp70 transcription by making the available pool of HSF1 active. Moreover it seems likely that in absence of the priming phosphorylation of HSF1 by ERK1/2, the secondary effect of the activated Gsk3β failed to inhibit HSF1 activity. This not only answers the initial apparent contradiction but further illustrates the essential involvement of both the Akt and ERK1/2 pathways in lowering of the Hsp70 levels by Resveratrol (Fig. 6).

17AAG is known to downregulate Bcr-Abl activity, however our study (Fig 5) along with a previous report [30] indicate that Resveratrol acts downstream of Bcr-Abl activity. This makes it tempting to propose that Resveratrol when used in combination may potentiate the action of the presently marketed Bcr-Abl inhibitors such as Imatinib mesylate,.

In essence, this present study provides a comprehensive investigation on the effect of Akt inactivation, Gsk3β activation and upregulation of ERK1/2 activity by Resveratrol in modulating the transcriptional efficacy of HSF1 and subsequent lowering of the Hsp70 levels in K562 cells.

## Supporting Information

Figure S1 Localization of Akt in untreated control K562 and Resveratrol-treated (6h) K562 cells.
(1.35 MB TIF)

## References

[1] Sreedhar AS, Csermely P (2004). Heat shock proteins in the regulation of apoptosis: new strategies in tumor therapy: a comprehensive review.. Pharmacol Ther.

[2] Ciocca DR, Calderwood SK (2005). Heat shock proteins in cancer: diagnostic, prognostic, predictive, and treatment implications.. Cell Stress Chaperones.

[3] Ma WW, Adjei AA (2009). Novel agents on the horizon for cancer therapy.. CA Cancer J Clin.

[4] Bagatell R, Whitesell L (2004). Altered Hsp90 function in cancer: a unique therapeutic opportunity.. Mol Cancer Ther.

[5] Parmiani G, Testori A, Maio M, Castelli C, Rivoltini L (2004). Heat shock proteins and their use as anticancer vaccines.. Clin Cancer Res.

[6] Whitesell L, Lindquist SL (2005). HSP90 and the chaperoning of cancer.. Nat Rev Cancer.

[7] Aggarwal BB, Van Kuiken ME, Iyer LH, Harikumar KB, Sung B (2009). Molecular targets of nutraceuticals derived from dietary spices: potential role in suppression of inflammation and tumorigenesis.. Exp Biol Med (Maywood).

[8] Sarkar FH, Li Y, Wang Z, Kong D (2009). Cellular signaling perturbation by natural products.. Cell Signal.

[9] Tao MH, Xu WH, Zheng W, Gao YT, Ruan ZX (2005). A case-control study in Shanghai of fruit and vegetable intake and endometrial cancer.. Br J Cancer.

[10] Pervaiz S, Holme AL (2009). Resveratrol: Its Biological Targets and Functional Activity.. Antioxid Redox Signal.

[11] Chakraborty PK, Mustafi SB, Ganguly S, Chatterjee M, Raha S (2008). Resveratrol induces apoptosis in K562 (chronic myelogenous leukemia) cells by targeting a key survival protein, heat shock protein 70.. Cancer Sci.

[12] Chakraborty PK, Mustafi SB, Raha S (2008). Pro-survival effects of repetitive low-grade oxidative stress are inhibited by simultaneous exposure to Resveratrol.. Pharmacol Res.

[13] Harikumar KB, Aggarwal BB (2008). Resveratrol: a multitargeted agent for age-associated chronic diseases.. Cell Cycle.

[14] Roccaro AM, Leleu X, Sacco A, Moreau AS, Hatjiharissi E (2008). Resveratrol exerts antiproliferative activity and induces apoptosis in Waldenstrom's macroglobulinemia.. Clin Cancer Res.

[15] Pocaly M, Lagarde V, Etienne G, Ribeil JA, Claverol S (2007). Overexpression of the heat-shock protein 70 is associated to imatinib resistance in chronic myeloid leukemia.. Leukemia.

[16] Quintas-Cardama A, Cortes J (2009). Molecular biology of bcr-abl1-positive chronic myeloid leukemia.. Blood.

[17] Druker BJ (2008). Translation of the Philadelphia chromosome into therapy for CML.. Blood.

[18] Kantarjian HM, Giles F, Quintas-Cardama A, Cortes J (2007). Important therapeutic targets in chronic myelogenous leukemia.. Clin Cancer Res.

[19] He B, Meng YH, Mivechi NF (1998). Glycogen synthase kinase 3β and extracellular signal-regulated kinase inactivate heat shock transcription factor 1 by facilitating the disappearance of transcriptionally active granules after heat shock.. Mol Cell Biol.

[20] Wang X, Grammatikakis N, Siganou A, Calderwood SK (2003). Regulation of molecular chaperone gene transcription involves the serine phosphorylation, 14-3-3 epsilon binding, and cytoplasmic sequestration of heat shock factor 1.. Mol Cell Biol.

[21] Westerheide SD, Anckar J, Stevens SM, Sistonen L, Morimoto RI (2009). Stress-inducible regulation of heat shock factor 1 by the deacetylase SIRT1.. Science.

[22] Kawauchi K, Ogasawara T, Yasuyama M, Ohkawa S (2003). Involvement of Akt kinase in the action of STI571 on chronic myelogenous leukemia cells.. Blood Cells Mol Dis.

[23] Nguyen TK, Rahmani M, Harada H, Dent P, Grant S (2007). MEK1/2 inhibitors sensitize Bcr/Abl+ human leukemia cells to the dual Abl/Src inhibitor BMS-354/825.. Blood.

[24] Guo F, Rocha K, Bali P, Pranpat M, Fiskus W (2005). Abrogation of heat shock protein 70 induction as a strategy to increase antileukemia activity of heat shock protein 90 inhibitor 17-allylamino-demethoxy geldanamycin.. Cancer Res.

[25] Solit DB, Basso AD, Olshen AB, Scher HI, Rosen N (2003). Inhibition of heat shock protein 90 function down-regulates Akt kinase and sensitizes tumors to Taxol.. Cancer Res.

[26] Frojdo S, Cozzone D, Vidal H, Pirola L (2007). Resveratrol is a class IA phosphoinositide 3-kinase inhibitor.. Biochem J.

[27] Aziz MH, Nihal M, Fu VX, Jarrard DF, Ahmad N (2006). Resveratrol-caused apoptosis of human prostate carcinoma LNCaP cells is mediated via modulation of phosphatidylinositol 3′-kinase/Akt pathway and Bcl-2 family proteins.. Mol Cancer Ther.

[28] Mukherjee S, Lekli I, Gurusamy N, Bertelli AA, Das DK (2009). Expression of the longevity proteins by both red and white wines and their cardioprotective components, resveratrol, tyrosol, and hydroxytyrosol.. Free Radic Biol Med.

[29] Rohde M, Daugaard M, Jensen MH, Helin K, Nylandsted J (2005). Members of the heat-shock protein 70 family promote cancer cell growth by distinct mechanisms.. Genes Dev.

[30] Puissant A, Grosso S, Jacquel A, Belhacene N, Colosetti P (2008). Imatinib mesylate-resistant human chronic myelogenous leukemia cell lines exhibit high sensitivity to the phytoalexin resveratrol.. Faseb J.

[31] Skorski T, Bellacosa A, Nieborowska-Skorska M, Majewski M, Martinez R (1997). Transformation of hematopoietic cells by BCR/ABL requires activation of a PI-3k/Akt-dependent pathway.. Embo J.

[32] Cotto JJ, Kline M, Morimoto RI (1996). Activation of heat shock factor 1 DNA binding precedes stress-induced serine phosphorylation. Evidence for a multistep pathway of regulation.. J Biol Chem.

[33] Chu B, Soncin F, Price BD, Stevenson MA, Calderwood SK (1996). Sequential phosphorylation by mitogen-activated protein kinase and glycogen synthase kinase 3 represses transcriptional activation by heat shock factor-1.. J Biol Chem.

[34] Coghlan MP, Culbert AA, Cross DAE, Corcoran SL, Yates JW (2000). Selective small molecule inhibitor s of glycogen synthase kinase-3 modulate glycogen metyabolism and gene transcription.. Chem Biol.

[35] Huang J, Gan Q, Han L, Li J, Zhang H (2008). SIRT1 overexpression antagonizes cellular senescence with activated ERK/S6K1 signaling in human diploid fibroblasts.. PLoS ONE.

[36] Zhang J (2006). Resveratrol inhibits insulin responses in a SirT1-independent pathway.. Bichem J.

