# Supplementary figures and images for: Modulation of Akt and ERK1/2 Pathways by Resveratrol in Chronic Myelogenous Leukemia (CML) Cells Results in the Downregulation of Hsp70
Source: PLoS One. 2010 Jan 14;5(1):e8719. doi: 10.1371/journal.pone.0008719 (PMC2806839; doi:10.1371/journal.pone.0008719)

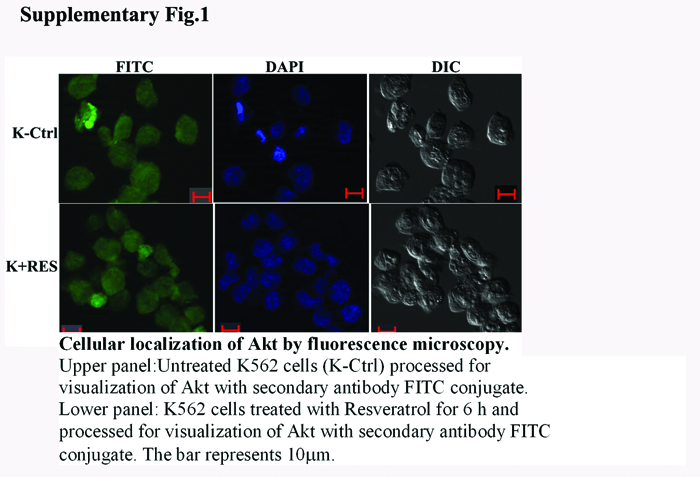

Supplement: Figure S1 — Localization of Akt in untreated control K562 and Resveratrol-treated (6h) K562 cells. (1.35 MB TIF) [file pone.0008719.s001.tif]
